# Experimental Infection of Potential Reservoir Hosts with Venezuelan Equine Encephalitis Virus, Mexico

**DOI:** 10.3201/eid1504.081008

**Published:** 2009-04

**Authors:** Eleanor R. Deardorff, Naomi L. Forrester, Amelia P. Travassos da Rosa, Jose G. Estrada-Franco, Roberto Navarro-Lopez, Robert B. Tesh, Scott C. Weaver

**Affiliations:** University of Texas Medical Branch, Galveston, Texas, USA (E.R. Deardorff, N.L. Forrester, A.P. Travassos da Rosa, J.G. Estrada-Franco, R.B. Tesh, S.C. Weaver); Comision Mexico–Estados Unidos para la Prevencion de la Fiebre Aftosa y Otras Enfermedades Exoticas de los Animales, Mexico City, Mexico (R. Navarro-Lopez)

**Keywords:** Zoonoses, vector-borne infections, bioterrorism agents and preparedness, encephalomyelitis virus, alphaviruses, transmission, Mexico, strains, wild rodents, research

## Abstract

In 1993, an outbreak of encephalitis among 125 affected equids in coastal Chiapas, Mexico, resulted in a 50% case-fatality rate. The outbreak was attributed to Venezuelan equine encephalitis virus (VEEV) subtype IE, not previously associated with equine disease and death. To better understand the ecology of this VEEV strain in Chiapas, we experimentally infected 5 species of wild rodents and evaluated their competence as reservoir and amplifying hosts. Rodents from 1 species (*Baiomys musculus*) showed signs of disease and died by day 8 postinoculation. Rodents from the 4 other species (*Liomys salvini*, *Oligoryzomys fulvescens*, *Oryzomys couesi,* and *Sigmodon hispidus*) became viremic but survived and developed neutralizing antibodies, indicating that multiple species may contribute to VEEV maintenance. By infecting numerous rodent species and producing adequate viremia, VEEV may increase its chances of long-term persistence in nature and could increase risk for establishment in disease-endemic areas and amplification outside the disease-endemic range.

## Abstract 2

Multiple wild rodent species can serve as amplifying reservoir hosts for virus subtype IE.

Venezuelan equine encephalitis (VEE) is a potentially fatal, reemerging disease in tropical America (the portions of North, South, and Central America between the tropics of Cancer and Capricorn) that can cause outbreaks involving hundreds of thousands of humans and equids. VEE virus (VEEV; *Togaviridae*: *Alphavirus*) strains are categorized as either epizootic (associated with equine disease and major epidemics of human disease through equine amplification), or enzootic (not known to cause equine disease). Most VEEV strains, both epizootic and enzootic, have been associated with human disease (*1*). VEEV is also of biodefense importance; it has been developed as a biological weapon, mainly because it is highly infectious by aerosol transmission and can infect humans with a relatively low dose (*2*).

During the mid-1990s, 2 epizootic equine outbreaks occurred in coastal Oaxaca and Chiapas states in Mexico; the causative agent was determined to be VEEV subtype IE (VEEV-IE), which was previously considered to be not virulent for equids (*1*). On the basis of the spread of a VEEV subtype IAB epizootic/epidemic through Mexico and into Texas in 1971 (*3*), the 1993 and 1996 outbreaks were considered to have the potential to spread to other regions of Mexico or the United States. To prevent, detect, and evaluate potential reemergence of this virus in the United States, we need to understand the factors that govern circulation and persistence of this virus in its enzootic foci and epizootic cycles.

Enzootic strains of VEEV are maintained naturally by transmission between mosquitoes of the subgenus *Culex (Melanoconion)* and wild rodents (*4*). These viruses are thought to circulate continuously among mosquitoes and their principal vertebrate amplifying hosts, whereas horses and humans are considered spillover, dead-end hosts not required for maintenance of the natural cycle. Several studies have shown that terrestrial mammals of 5 genera (*Didelphis*, *Oryzomys*, *Proechimys*, *Sigmodon*, and *Zygodontomys*) are susceptible to VEEV-IE infection; they develop viremia sufficient to infect mosquito vectors, yet they usually survive infection (*5*–*10*).

Several species of wild rodents captured in coastal Chiapas have VEEV-specific antibodies (*11*). To address which of these species are likely to play a role as reservoir and/or amplification hosts, we captured rodents from 5 genera (*Baiomys*, *Liomys*, *Oligoryzomys*, *Oryzomys*, and *Sigmodon*) and transported them to the laboratory for experimental infection studies. Our goals were to evaluate the role of various vertebrate species in VEEV-IE maintenance and to help interpret seroprevalence data gathered in the field.

## Materials and Methods

### Animals

During October 2007, wild rodents of 5 species were collected from coastal Chiapas, Mexico: *Baiomys musculus* (southern pygmy mouse), *Liomys salvini* (Salvins spiny pocket mouse), *Oligoryzomys fulvescens* (fulvus pygmy rice rat), *Oryzomys couesi* (Coues’ rice rat) and *Sigmodon hispidus* (hispid cotton rat). All animals were captured from an overgrown field surrounding a stream in Mapastepec municipality, ≈2 km from the Pacific coast (15.413°N and 093.070°W) by using live-capture Sherman traps (H.B. Sherman Traps, Tallahassee, FL, USA). Species identification was based initially on morphologic features (*12*) and later confirmed genetically by using cytochrome-B gene sequences (*13*). Animals were housed individually and transported in Taconic Transit Cages (Taconic Farms, Inc., Hudson, NY, USA) to the Animal Biosafety Level 3 Facility at the University of Texas Medical Branch in Galveston, Texas, USA. Animals were captured under permit number SGPA/DGVS/03858/07 Julio 2 de 2007, issued to J.G.E.-F.; all studies were approved by the University of Texas Medical Branch Institutional Animal Care and Use Committee.

### Virus and Infection

Immediately before rodents were inoculated with virus, a baseline serum sample was taken from each rodent for subsequent antibody assays. For inoculation we used VEEV strain MX01-22 (subtype IE). This strain had been isolated in 2001 from a sentinel hamster in coastal Chiapas, Mexico, and passaged once in Vero cells to generate a sufficient volume of high-titer virus for experimentation. We chose this strain because it is the most recent low-passage isolate of VEEV from the outbreak area and because transmission of this strain by VEEV mosquito vector species from this area has been studied (*14*,*15*). Additionally, this strain is genetically highly similar to the equine-virulent strains that were isolated during the 1993 outbreak (*11*) and caused encephalitis in horses (R. Bowen, pers. comm.).

All animals were inoculated subcutaneously in the right thigh with 3.2 log_10_ PFU of virus, a dose that approximates the maximum amount of VEEV transmitted by a mosquito bite (*16*). After inoculation, all animals were weighed daily for 1 week and observed for signs of illness for 2 weeks.

### Viremia Assays

Blood was collected daily for the first 7 days after inoculation, then on days 10, 14, 28, 42, and 66. After the animals were anesthetized with inhaled isoflurane, retroorbital sinus blood was collected in heparinized glass capillary tubes and transferred to 5 volumes of phosphate-buffered saline (PBS). Erythrocytes were removed by centrifugation to yield an ≈1:10 dilution of serum, which was stored at –80ºC. Viremia titers were determined by plaque assay on Vero cells (*17*).

Necropsy was performed on all animals, and tissues were frozen at –80ºC. Using a TissueLyser (QIAGEN Inc, Valencia, CA, USA), we homogenized ≈2–10 mg of tissue in minimal essential medium (Eagle) supplemented with 20% fetal bovine serum, l-glutamine, penicillin, streptomycin, gentamicin, and fungizone. Tissue virus titers were determined by plaque assay on Vero cells.

### Antibody Assays

To detect VEEV-IE–specific antibodies, we performed hemagglutination inhibition assays (*17*) using antigen derived from the same VEEV strain used for infection (MX01-22) as well as from 3 other arboviruses: Eastern equine encephalitis virus (TenBroeck strain), West Nile virus (strain 385-99), and St. Louis encephalitis virus (strain TBH28). Briefly, 4–8 units of hemagglutinin antigen were reacted with heat-inactivated test serum in various concentrations in PBS. Failure to hemagglutinate goose erythrocytes was considered a positive result. Antibody titers were confirmed by plaque reduction neutralization tests (*17*). Test serum samples were serially diluted in PBS and heat inactivated at 56°C for 1 h, then mixed with ≈100 PFU of virus and incubated at 37°C for 1 h. The mixture was inoculated onto Vero cells. Dilutions resulting in >80% reduction in virus titer were considered positive; titers were reported as the reciprocal of the endpoint dilution.

## Results

### Clinical Responses and Survival Rates

**Figure 1:**
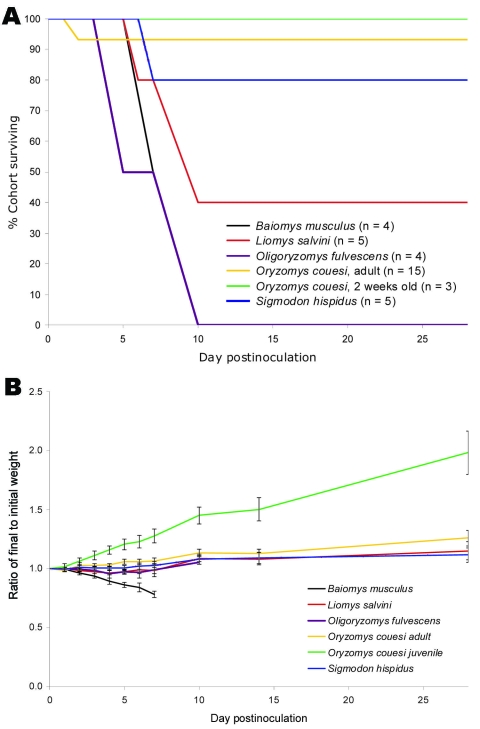
Survival rates and weight change of wild rodents from Chiapas, Mexico, after experimental infection with 3 log_10_ PFU of Venezuelan equine encephalitis virus subtype IE, strain MX01-22. A) Survival rates. Black and yellow lines represent animals whose brains yielded live virus after necropsy. Red, green, blue, and purple lines indicate animals whose death was attributed to manipulation and/or stress, not to VEEV infection. B) Weight change. Mean cohort weight (grams) divided by mean cohort starting weight (day 0). Weight gain or loss was used as an indicator of disease. Only *Baiomys musculus* rodents showed weight loss during acute infection. Data for days 42 and 66 (not shown) did not differ significantly from that for day 28. Error bars indicate SEM.

Of the 5 rodent species examined, only those of species *B. musculus* showed signs of disease with neurologic manifestations. These animals began to exhibit tremor, lethargy, dehydration, hunching, and staggering during days 4–6 postinoculation. By day 8, all 4 (100%) of these *B. musculus* rodents had died or were euthanized after becoming moribund (Figure 1, panel A). Rodents of this species were the only ones that lost body weight after inoculation (average 22% loss; Figure 1, panel B).

No animal from the other 4 species exhibited weight loss or outward signs of illness after inoculation. Most of these rodents survived until the end of the experiment, day 66 postinoculation. However, during the first 2 weeks after inoculation, 9 animals died without weight loss or signs of illness. These animals did not have high levels of virus in their tissues (Table) and are considered to have died of stress from daily manipulations rather than of VEEV infection. To address this possibility, a subcohort of 2 *L. salvini* and 3 *O. fulvescens* rodents, the 2 species that had had the most manipulation-related deaths, were inoculated and observed for 15 days without daily blood sampling. All 5 animals survived with little to no illness; they were found to have seroconverted by day 15 (reciprocal mean titer = 2.7 ± 2.3 log_10_, standard error) and remained seropositive through day 42 (3.0 ± 2.9 log_10_). Similar deaths of wild rodents in the absence of an infectious cause have been encountered previously (*10*).

**Table:** Viremia in rodents that died 1–14 days after inoculation with 3.2 log_10_ PFU of Venezuelan equine encephalitis virus subtype IE strain MX01-22*

Rodent genus	dpi†	Tissue virus content (log_10_ PFU/g)†					
		Brain	Heart	Spleen	Kidney	Liver	Lung
*Oryzomys*	2	1.8	2.7	3.3	2.0	3.2	0
*Oligoryzomys*	4	0	0	4.0	0	0	0
	6	0	0	3.4	0	0	0
*Baiomys*	6	3.2	5.0	5.0	4.7	4.9	3.9
	7	4.6	3.2	4.2	0	4.3	4.3
	7	2.0	2.0	3.0	3.4	2.6	4.1
	8	5.0	3.0	5.7	5.0	1.9	5.0

*Not shown are 3 *Liomys salvini*, 2 *Oligoryzomys fulvescens,* and 1 *Sigmodon hispidus* rodents. These animals died on days 5–10 postinoculation and showed no detectable virus in any organs tested. dpi, days postinoculation. †Limit of detection was 1 plaque in150 μL homogenate. Tissue sample weight varied between 0.002 and 0.01 grams.

### Virus Titers

**Figure 2:**
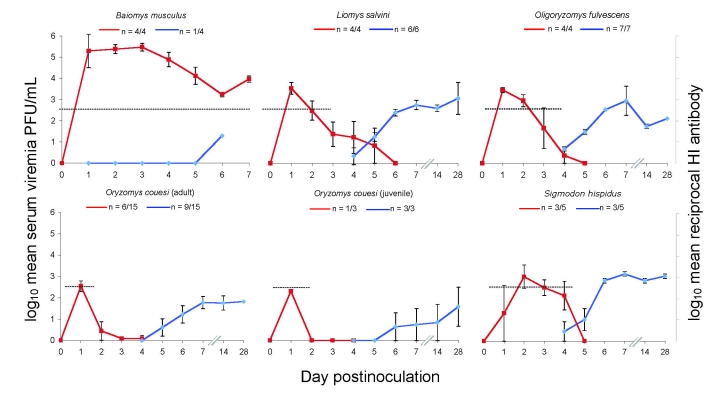
Mean viremia profile (red lines) and mean hemagglutination inhibition (HI) antibody profile (blue lines) of 5 species of wild rodents after experimental infection with 3 log_10_ PFU of Venezuelan equine encephalitis virus type-IE, strain MX01-22. Black dashed lines indicate approximate mosquito infection viremia threshold for the enzootic vector *Culex (Melanoconion) taeniopus*. Fractions represent proportion of total cohort that had measurable response. Data for days 42 and 66 (not shown) did not differ significantly from data for day 28. Error bars indicate SEM.

Of 35 animals tested, 22 (comprising all 5 species) had measurable virus levels during the first week after inoculation (limit of detection was 1.5 log_10_ PFU/mL) (Figure 2). Viremia (>2.7 log_10_ PFU/mL) developed in all (100%) *O. fulvescens,* *L. salvini*, and *B. musculus* rodents and lasted as long as 4, 5, and 8 days, respectively. Conversely, detectable viremia developed in only 60% of the cohort of *S. hispidus* rodents (3/5 animals), lasting as long as 4 days, and in only 39% of the *O. couesi* cohort (7/18 animals), lasting as long as 2 days.

In the cohorts of *L. salvini*, *O. fulvescens,* and *O. couesi* rodents, maximum viremia occurred on day 1 postinoculation; mean titers were 3.4 ± 0.6 (SEM), 3.3 ± 0.2, and 2.5 ± 0.6 log_10_ PFU/mL, respectively (Figure 2). In *S. hispidus* rodents, the cohort peak viremia occurred on day 2 postinoculation; mean was 2.9 log_10_ ± 0.9. In the cohort of *B. musculus* rodents, peak viremia occurred on day 3; mean was 5.5 ± 0.4 PFU/mL (Figure 2).

### Antibody Responses

Of the 40 animals used in this study, only 1 (*S. hispidus*) was found to have preexisting VEEV antibodies. This animal had a hemagglutination inhibition reciprocal antibody titer of 2.8 log_10_ on day 0 and 2.2 log_10_ on day 6, when it died during anesthesia and blood collection. For rodents of all 4 surviving species, antibodies were detectable by day 5 and lasted through the end of the experiment (Figure 2).

### Age Dependence

An unanticipated cohort of 3 juvenile rodents (*O. couesi*) provided an opportunity to examine whether age affected outcome of VEEV infection. The species of these 3 animals was initially identified as *O. fulvescens* but later determined, based on cytochrome-B gene sequencing, to be juvenile *O. couesi* (*13*). Age at infection was ≈2 weeks, determined on the basis of growth of 3 litters of *O. couesi* rodents born in captivity.

No differences were found between the juvenile and the adult *O. couesi* rodents in terms of survival rates, viremia levels, or antibody responses (Figures 1, 2). Viremia was detected in 1 (33%) of 3 juvenile and 6 (40%) of 15 adult *O. couesi* rodents. Mean maximum viremia was 2.3 log_10_ PFU/mL for the juveniles and 2.6 ± 0.6 log_10_ PFU/mL for the adults. No viremia was detected after day 1 for either juveniles or adults, except for 1 adult that had a titer of 2.6 log_10_ on day 2. Antibody responses were inconsistent among animals from both groups. Several animals from each group showed weak antibody responses of short duration, delayed onset, or both, after having no detectable viremia.

## Discussion

### Reservoir Status and Potential

Of the 5 species of rodents evaluated in this study, only *S. hispidus* rodents have been included in previous experimental VEEV infection studies. In Panama (*10*) and Florida (*5**,**7*), *S. hispidus* rodents are considered to be competent, mostly disease-resistant reservoir hosts for disease caused by sympatric VEE complex alphaviruses. In 2007, Carrara et al. (*7*) infected 3 geographically distinct populations of *S. hispidus* rodents with 2 enzootic VEEV strains and found that only the population from a VEE complex alphavirus–endemic region (Florida) survived infection; cohorts from the 2 non–virus-endemic populations succumbed to disease. For this reason, we used a sympatric VEEV strain for our studies.

In addition to *S. hispidus* rodents, 3 other species (*Proechimys semispinosus, Zygodontomys microtinus*, and *Oryzomys capito*) had viremia sufficient to infect at least some mosquito vectors and survive after inoculation with sympatric strains of VEEV (*8*–*10*). Our results support the hypothesis that enzootic VEEV selects for resistance to disease in its sympatric reservoir host populations (*10*).

Several field studies in Mexico have reported VEEV-specific antibodies in a variety of wild vertebrate species. Aguirre et al. (*18*) found 7 species of wild mammals and 17 species of wild birds that were seropositive against VEEV-IE in 1992. In the same area from which the animals for our study were captured, VEEV-neutralizing antibodies were detected in wild *S. hispidus, Oryzomys alfaroi*, and *Didelphis marsupialis* rodents (*11*). In an extensive field study in southern Mexico during the 1960s, Scherer et al. (*6*) found 29 species of wild birds, 10 genera of terrestrial mammals, and 3 genera of bats with serologic evidence of natural VEEV infection. Evidence of similar broad host ranges of VEEV has been found in coastal Guatemala, where 7 genera of terrestrial mammals and 11 species of birds had VEEV-specific antibodies (*19*). After the 1971 epidemic of VEEV-IAB that started in Central America and reached southern Texas, extensive field studies were conducted to determine whether the virus would or could establish a new enzootic focus (*20*). In that study, mammals of 10 genera had VEEV-specific antibodies. In 2 follow-up studies in which wild mammals and wild birds were infected with a strain of VEEV-IB isolated during the outbreak, viremia and mortality rates for rodents were high (*21**,**22*). In a longitudinal field study performed concurrent with the study reported here, seroprevalance for wild rodents was found to be much lower than previously found for this area (*11*).

### Viremia and Immunologic Response

All 5 of the species tested produced viremia titers sufficient to infect the proven enzootic mosquito vector *Cx. (Mel.) taeniopus.* Of these 5 species, the lowest and shortest lasting viremia was found in *O. couesi* rodents; however, even these reached levels that are considered adequate to infect a proportion of *Cx. (Mel.) taeniopus* (*23*). The other 4 species all exhibited viremia titers well above the minimum infection threshold for this vector. Therefore, assuming that they are bitten by *Cx. (Mel.) taeniopus* mosquitoes*,* which are known to be universal feeders and have been recently found in higher numbers than previously reported in the area where these animals were captured, all 5 species we studied should be able to infect this mosquito (*11**,**24*).

**Figure 3:**
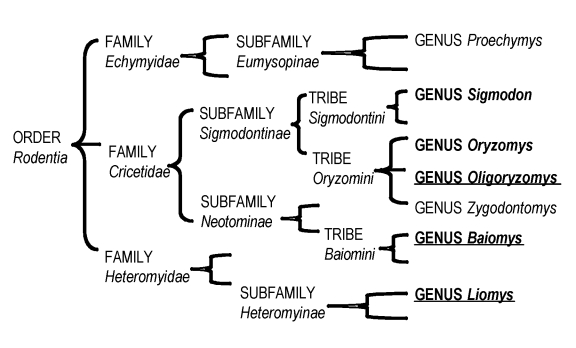
Relatedness of 7 wild rodent genera that have been experimentally evaluated for suitability as amplifying hosts in enzootic transmission cycles of Venezuelan equine encephalitis virus. The 5 genera included in this study are presented in **boldface;** the 3 novel genera are underlined**.**

The uniform susceptibility of *B. musculus* rodents to VEE disease was an unexpected result and appeared to contradict the hypothesis that VEEV circulation selects for resistance to disease in wild rodents. This difference is evidently not reflective of the taxonomic relatedness of these 5 species (Figure 3). A different potential explanation is the lack of temporal overlap of activity between *B. musculus* rodents and the enzootic vector, *Cx. (Mel.) taeniopus. Baiomys* spp. rodents are diurnally active (*12*), but *Cx. (Mel.) taeniopus* mosquitoes are nocturnal feeders (*24*,*25*). Although the rodents and mosquito vectors coexist spatially, they are not active at the same time of day, which may limit their contact. This lack of contact time may preclude the selection for resistance to VEE that is manifested in the other 4 rodent species, which are nocturnal and presumably regularly exposed to bites from this vector. Experimental infection of other diurnal species from the study area, or similar studies in another VEE-endemic area, could be used to test this hypothesis. Of the 5 species, *B. musculus* rodents were the only species not encountered in previous capture-and-release studies; however, because of the severity of disease in this species, seropositive individuals would be unlikely to survive (and thereby be caught) in the wild.

We ended our study at 66 days postinoculation for the original cohort and 42 days postinoculation for the subcohorts of *L. salvini* and *O. fulvescens* that survived*.* The antibody responses for all animals that developed measurable viremia persisted through the end of the experiment. The only exception was several *O. couesi* animals that did not develop viremia but did demonstrate brief, low-titer (<1.6 log_10_) antibody responses. Wild rodents have been shown to remain seropositive for as many as 6 months postinoculation with VEEV (*7*). For some species with short life spans in the wild, this antibody response is tantamount to life-long immunity offering protection against reinfection and affording more opportunity for the animal to reproduce.

### Ecological Implications

Although the ability of laboratory experimentation to elucidate natural processes is limited, data gathered in the laboratory are sometimes more complete and detailed than field data. In this study, 5 of the most commonly captured rodent species in coastal Chiapas, Mexico, were evaluated for their ability to participate in the natural transmission cycle of enzootic VEEV-IE*. S. hispidus* and *O. capito* rodents have previously been implicated in amplification of other VEE subtypes, ID, IE, and II (*7*–*9*), but the other 3 species (*B. musculus, L. salvini*, and *O. fulvescens*) had been studied little or not at all. Rodents of all 5 species developed viremia titers sufficient to infect the enzootic mosquito vector, *Cx. (Mel.) taeniopus*. However, only 4 of the 5 species survived infection with the potential to reproduce, a trait considered critical for true reservoir status in that it avoids population declines that might jeopardize long-term virus circulation.

History has shown that an outbreak of highly virulent VEEV in southern Mexico can easily and rapidly spread into the United States, as it did in 1971. Therefore, a better understanding of VEEV ecology in Mexico is essential for assessing the risk for widespread disease. Our results support the conclusions of Scherer et al. (*6*) that VEEV has a wide range of mammalian hosts that may participate in the natural transmission cycle. This strategy may be an adaptive one that affords greater population stability than does specialization for 1 amplifying host species. By being able to infect numerous rodent species and produce adequate viremia for mosquito transmission, VEEV may increase its chances of long-term persistence in nature when weather or environmental conditions affect some but not all reservoir host populations. This ability could also increase the risk for endemic establishment as well as amplification when outbreaks spread outside their disease-endemic range.
